# Supplementary material for: Sex differentiation in grayling (Salmonidae) goes through an all-male stage and is delayed in genetic males who instead grow faster
Source: Sci Rep. 2017 Nov 3;7:15024. doi: 10.1038/s41598-017-14905-9 (PMC5670243; doi:10.1038/s41598-017-14905-9)

Supplementary Material

Sex differentiation in grayling (Salmonidae) goes through an all-male stage and is delayed in genetic males who instead grow faster

Diane Maitre[1*], Oliver M. Selmoni[1*], Anshu Uppal[1*], Lucas Marques da Cunha[1], Laetitia G. E. Wilkins[1], Julien Roux[1,2], Kenyon B. Mobley[1], Isabelle Castro[1], Susanne Knörr[3#], Marc Robinson-Rechavi[1,2#], Claus Wedekind[1#]

[1] *Department of Ecology and Evolution, Biophore, University of Lausanne, 1015 Lausanne, Switzerland*

[2] *Swiss Institute of Bioinformatics, 1015 Lausanne, Switzerland*

[3] *Aquatic Ecology and Toxicology Group, Center of Organismic Studies, University of Heidelberg, Heidelberg, Germany*

* *equal contributors*

[#] *shared senior authors*

Present addresses:
O. Selmoni: Swiss Federal Institute of Technology (EPFL), Bâtiment GC, 1015 Lausanne, Switzerland
L. Wilkins: Department of Environmental Sciences, Policy and Management, 130 Mulford Hall #3114, University of California, Berkeley, CA 94720, USA
K. Mobley: Max-Planck Institute for Evolutionary Biology, Department of Evolutionary Ecology, August Thienemann Str. 2, 24306 Plön, Germany
J. Roux: Department of Biomedicine, University of Basel, Hebelstr. 20, 4031 Basel, Switzerland
I. Castro: Veterinäramt Zürich, 8090 Zürich, Switzerland

Correspondence: Claus Wedekind, claus.wedekind@unil.ch, orcid.org/0000-0001-6143-4716

Supplementary Table S1. Experimental and biological condition.

ID	TP	TR	SI	Cell	Run	Sex	ng/ul (ND)	A260	A280	260/280	260/230	ng/ul (FA)	RQN
1	1	C	93	1-A4	1	M	62.06	1.551	0.732	2.12	1.96	64.56	9.7
2	1	C	94	1-D9	1	M	55.8	1.395	0.595	2.34	1.69	53.2	10
3	1	C	95	3-A1	3	M	55.09	1.377	0.696	1.98	1.13	38.85	9
4	1	C	96	3-G10	3	M	77.68	1.942	0.948	2.05	1.73	86.4	8.6
5	1	C	97	2-D4	2	M	132.16	3.304	1.544	2.14	2.1	108.29	9.8
6	1	E	93	1-D4	1	M	84.43	2.111	1.031	2.05	2.36	69.83	10
37	1	E	94	2-B10	2	M	123.14	3.079	1.462	2.11	1.98	88.29	9.7
8	1	E	95	3-F4	3	M	170.61	4.265	2.01	2.12	2	79.97	9.1
9	1	E	96	3-A9	3	M	99.18	2.479	1.192	2.08	2.09	51.4	9.7
65	1	E	97	1-C12	1	M	57.4	1.435	0.676	2.12	1.45	122.38	9.9
66	2	C	93	2-D7	2	M	92.63	1.853	0.916	2.02	0.9	78.83	8.9
12	2	C	94	3-D5	3	F	65.39	1.635	0.849	1.92	1.33	40.3	9.2
13	2	C	95	1-A11	1	M	54.22	1.355	0.645	2.1	2.25	56.93	9.5
14	2	C	96	1-H4	1	M	30.82	0.771	0.324	2.37	2.76	36.99	10
15	2	C	97	1-G5	1	M	34.27	0.857	0.421	2.04	1.17	35.56	9
16	2	E	93	3-E3	3	M	102.5	2.563	1.209	2.12	2.61	63.84	9.5
67	2	E	94	2-A7	2	M	86.07	2.152	1.075	2	1.3	48	7
18	2	E	95	2-G1	2	M	77.09	1.927	0.931	2.07	1.07	57.25	8.5
19	2	E	96	1-F6	1	M	42.95	1.074	0.489	2.2	2.64	45.94	9.9
20	2	E	97	2-H4	2	M	64.68	1.617	0.75	2.15	2.44	48.54	9.1
21	3	C	93	3-B8	3	M	103.1	2.578	1.247	2.07	1.6	55.83	8.1
22	3	C	94	3-C6	3	M	113.69	2.842	1.344	2.11	1.85	74.5	8.3
23	3	C	95	2-C3	2	M	151.11	3.778	1.751	2.16	2.61	115.08	8.1
24	3	C	96	2-F8	2	M	107.9	2.698	1.291	2.09	1.48	68.65	7.8
25	3	C	97	2-C8	2	M	287.46	7.187	3.38	2.13	1.8	202.69	8.7
26	3	E	93	2-E1	2	M	93.17	2.329	1.084	2.15	2.18	74.68	8.4
27	3	E	94	3-C3	3	M	137.06	3.427	1.629	2.1	2.42	86.62	9.2
28	3	E	95	3-E10	3	M	93.37	2.334	1.151	2.03	2	58.83	8
61	3	E	96	1-B4	1	M	47.93	1.198	0.602	1.99	3.04	79.19	8.5
30	3	E	97	1-B9	1	M	135.33	3.383	1.621	2.09	1.98	145.31	9.1
31	1	C	93	1-A3	1	F	60.14	1.505	0.815	1.84	0.98	40.86	9.5
64	1	C	94	1-D8	1	F	64.95	1.624	0.821	1.98	1.41	87.3	9.6
33	1	C	95	3-A2	3	F	90.13	2.253	1.062	2.12	1.63	63.04	9.5
34	1	C	96	3-G9	3	F	38.26	0.956	0.425	2.25	2.13	65.33	8.6
35	1	C	97	2-D3	2	F	94.51	2.363	1.134	2.08	1.84	98.04	9.8
36	1	E	93	1-D2	1	F	53.39	1.335	0.602	2.22	1.48	49.47	9.7
68	1	E	94	2-B11	2	M	94.86	2.372	1.173	2.02	1.2	59.4	7.5
38	1	E	95	3-F2	3	F	129.17	3.229	1.555	2.08	1.78	69.2	10
39	1	E	96	3-A7	3	F	106.58	2.664	1.233	2.16	1.68	78.38	9.1
63	1	E	97	1-C8	1	F	81.43	2.036	0.99	2.06	1.8	101.32	9.3
41	2	C	93	2-D5	2	F	70.13	1.753	0.854	2.05	1.27	63.17	9
42	2	C	94	3-D4	3	F	65.29	1.632	0.798	2.04	1.83	45.53	8.9
43	2	C	95	1-A7	1	F	41.67	1.042	0.517	2.02	2.26	44.24	9.4
44	2	C	96	1-H5	1	F	31.3	0.783	0.362	2.16	1.71	38.48	10
45	2	C	97	1-G8	1	F	41	1.025	0.462	2.22	1.88	32.23	10
46	2	E	93	3-E1	3	F	97.64	2.441	1.238	1.97	2.36	65.55	9.9
47	2	E	94	2-A5	2	F	79.06	1.976	0.903	2.19	1.65	60.85	9.1
48	2	E	95	2-F10	2	F	74.86	1.871	0.893	2.09	2.4	58.88	9.2
49	2	E	96	1-F4	1	F	45.81	1.145	0.558	2.05	2.18	48.67	10
50	2	E	97	2-H3	2	F	71.38	1.785	0.857	2.08	2.01	59.54	8.7
51	3	C	93	3-B9	3	F	84.79	2.12	0.991	2.14	1.96	52.75	9.8
52	3	C	94	3-C7	3	F	126.02	3.151	1.484	2.12	1.45	81.82	8.8
53	3	C	95	2-C4	2	F	124.85	3.121	1.458	2.14	2.04	96.53	8.3
54	3	C	96	2-F5	2	F	80.66	2.016	0.941	2.14	1.84	64.31	7.2
55	3	C	97	2-C6	2	M	225.2	5.63	2.646	2.13	2.15	172.89	8.3
56	3	E	93	2-E2	2	M	166.02	4.15	1.963	2.11	2.27	132.32	8
57	3	E	94	3-C1	3	F	115.68	2.892	1.428	2.03	1.55	94.42	8.5
58	3	E	95	3-E11	3	F	99.85	2.496	1.221	2.04	2.62	80.9	6.1
59	3	E	96	1-B5	1	F	84.86	2.121	1.115	1.9	1.53	57.42	8.9
60	3	E	97	1-B11	1	F	123.96	3.099	1.452	2.13	1.86	130.93	8.2

ID = sample identifier, TP = time point of sampling (1 = embryo-stage, 2 = hatching-day, 3 = first-feeding-stage), TR = treatment (C=control, E=EE2), SI= sibgroup, Cell = cell in 96 well plate of RNA extraction, Run = Run of RNA extraction, ng/uL(ND) = concentration of RNA by Nanodrop, A260 = absorbance at 260 nm, A280 = absorbance at 280 nm, 260/280 = ratio of absorbances, 260/230 = ratio of absorbances, ng/ul(FA) = concentration of RNA by Fragment Analyzer, RQN=RNA quality index.

Supplementary Table S2. Sequencing statistics of samples.

ID	Lane	Yield (Mbases)	Raw Reads Nb	Mean Quality Score
1	2	8951	94'310'746	35.23
2	6	7728	80'727'600	35.32
3	2	8434	89'100'552	35.22
4	5	7202	74'249'340	35.51
5	1	9339	98'912'292	35.03
6	3	7986	84'077'272	35.08
8	6	9476	100'430'984	35.08
9	5	7159	73'602'988	35.74
12	7	8590	88'975'372	35.49
13	8	8373	87'518'388	35.27
14	5	5453	57'482'172	34.73
15	6	8376	89'584'502	34.81
16	3	6814	72'385'354	34.85
18	5	9822	101'412'350	35.5
19	5	8864	93'949'962	35.2
20	1	9948	105'392'198	35.04
21	5	8917	94'149'480	35.19
22	7	8797	91'463'958	35.25
23	7	10727	111'651'906	35.36
24	7	6311	65'582'824	35.33
25	8	10682	111'546'226	35.21
26	8	4978	52'219'004	35.1
27	3	10277	108'106'904	35.06
28	6	8803	92'053'888	35.19
30	5	6081	62'680'856	35.38
31	7	7467	77'588'734	35.27
33	6	8544	89'545'780	35.28
34	3	8823	92'375'346	35.23
35	1	8618	91'765'202	34.81
36	4	10423	110'533'770	34.97
37	4	7630	81'074'258	34.91
38	7	7912	82'886'272	35.05
39	6	7781	83'083'296	34.99
41	4	9518	100'729'174	35.13
42	6	8168	86'722'276	35.14
43	6	9226	97'628'958	35.13
44	2	9512	100'112'566	34.99
45	8	8145	85'152'984	35.28
46	3	9012	95'051'902	35.03
47	5	8993	95'242'488	35.12
48	1	9169	96'915'018	34.94
49	6	7839	82'485'106	35
50	2	8872	93'464'052	35.11
51	2	8775	92'371'168	35.05
52	1	8920	94'572'332	34.89
53	3	10503	110'538'692	35.04
54	5	9787	100'738'916	35.45
55	1	8451	89'566'368	34.97
56	5	8874	94'121'488	35.02
57	4	9180	96'847'664	35.12
58	2	8642	90'872'204	35.25
59	5	9067	95'871'206	35.26
60	6	9946	106'909'132	34.43
61	8	9395	98'030'932	35.22
64	8	10255	107'142'268	35.3
65	4	9710	103'383'790	34.82
66	5	5254	55'447'016	35.35
67	4	8567	90'719'714	35.01
68	6	8300	89'764'786	34.51
63	6	9629	101'343'034	35.04

Table resuming the read numbers and quality of the RNA libraries. ID= sample identifier, Lane= lane of sequencing, Yield= number of Megabases sequenced, Raw Reads Nb= number of 100 bp reads sequenced, Mean Quality Score= mean Phred quality score across all sequenced bases.

Supplementary Table S3. Samples used to produce the transcriptome assembly.

	control male	control female	EE2 male	EE2 female
embryo stage	03, 02	64, 31	06, 08	63, 36
hatching day	13, 15	45, 43	16, 19	49, 46
first feeding	22, 21	51, 53	61, 27	58, 59

The samples used for assembly are sorted by developmental stage, sex and treatment. All duplicated reads within a sample were filtered before assembly. Each combination of developmental stage, treatment and sex was represented by two samples. Embryo stage: 21 *dpf*; Hatching day: *31dpf*; First feeding: 52 *dpf*.

Supplementary Figure S1. Example of electrophoretic migration of the PCR products used to determine the genotypic sex of individuals. Blue and red labels indicate males and females, respectively.

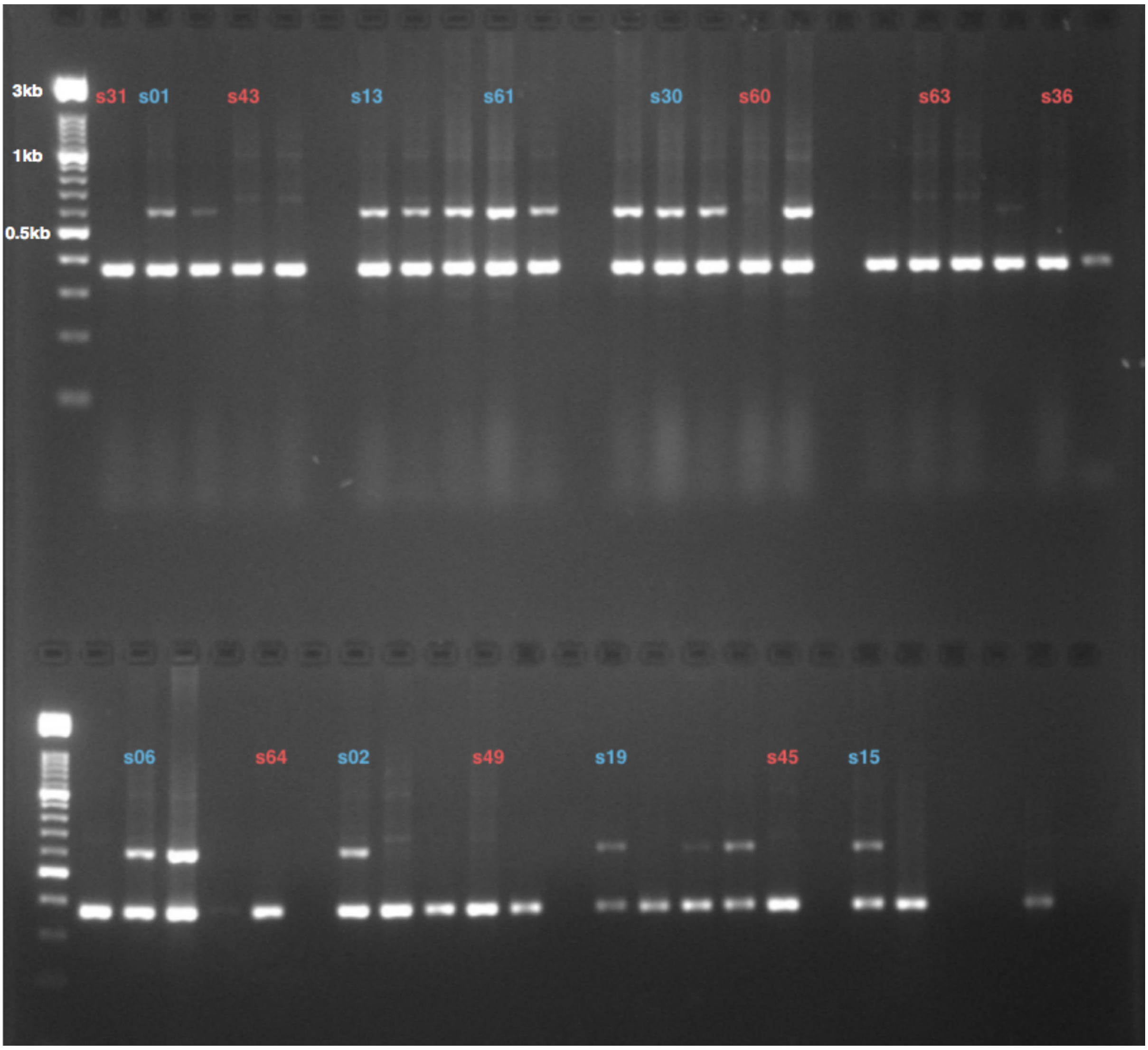

Supplementary Figure S2. Expression density curves. Each line represents the density distribution of the expression value (log2(cpm)) for a specific sample. After filtering and normalization processing, the density distribution of the expression values appears similar between the different samples.

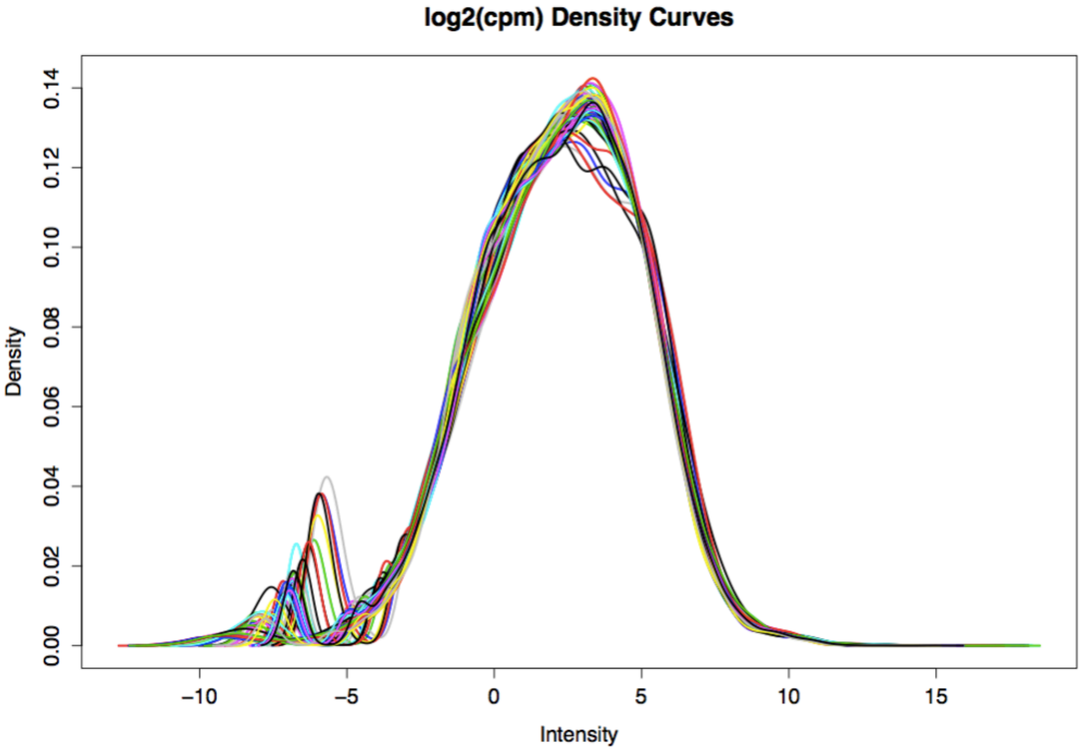

Supplementary Figure S3. Differential gene expression between control males and control females at embryo stage (21 *dpf*). The upper graph shows the average expression and the log-fold change in expression between the two developmental stages for each gene. Each dot is a gene. If the dot is red, the gene is differentially expressed (q<0.15). If the log fold change is positive, the expression is stronger in the first term of the comparison (here in males), if negative in the second term (here in females). In total 15 genes were found differentially expressed (q<0.15) between control males and control females at embryo stage. The graphs on the bottom show the significance of the comparisons by analyzing the frequency distribution of the p-values. If there is no significant difference between gene expression of all gene between the two conditions, then the frequency distribution of the p-values will appear flat (i.e. as expected under the null hypothesis: no difference between condition) on the graph on the bottom left. This same result will appear on the bottom right graph as the observed p-values being identical to the expected by chance (i.e. following the red line). If there are genes that are truly differentially expressed between the conditions, the distribution will skew towards the 0 limit of the p-value axis in the bottom left graph (as in this case). In this case, the smallest observed p-values will be smaller than the smallest expected by chance p-values in the bottom right graph.

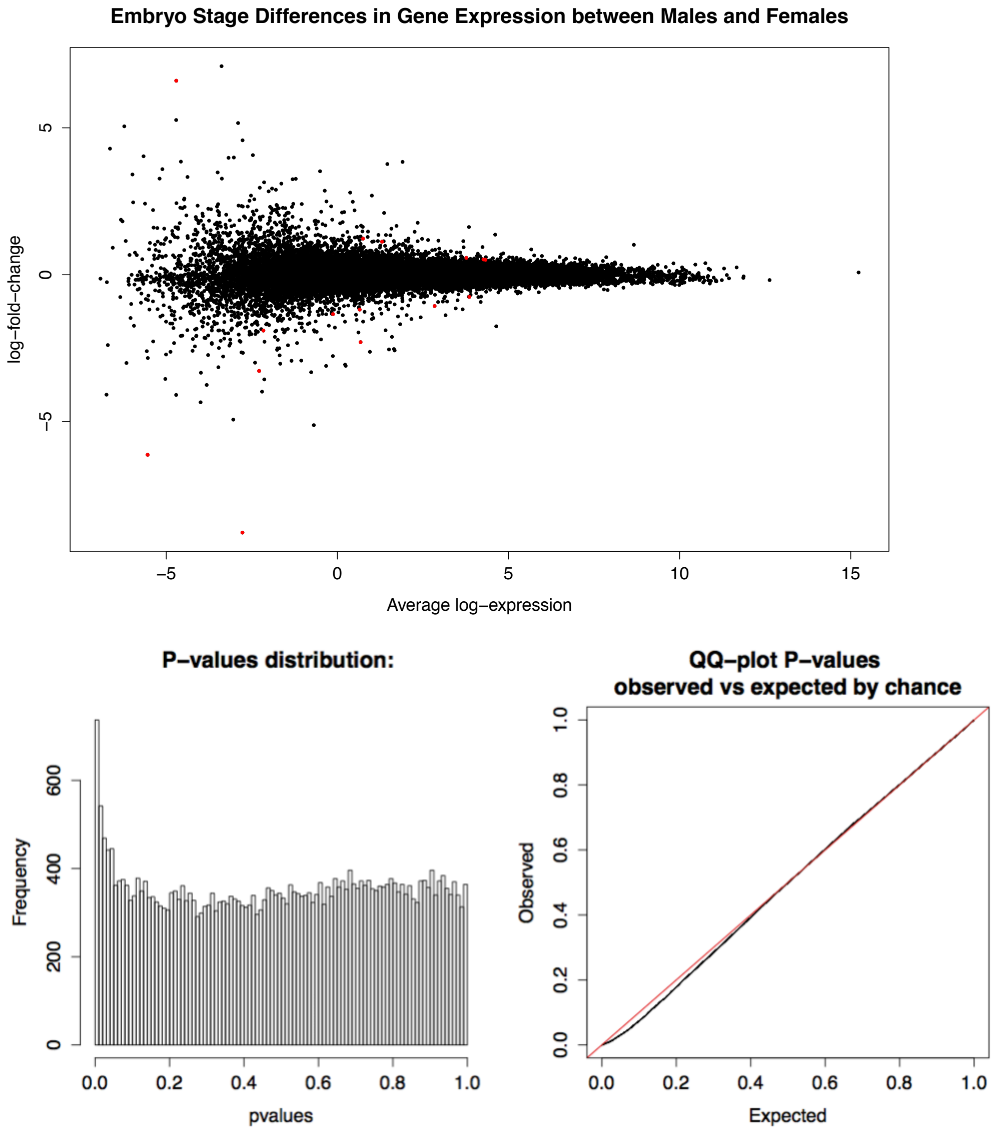

Supplementary Figure S4. Differential gene expression between control males and control females at hatching day (31 *dpf*). In total 25,372 genes were found differentially expressed (q<0.15) between control males and control females at hatching day. See Figure S4 for further explanations.

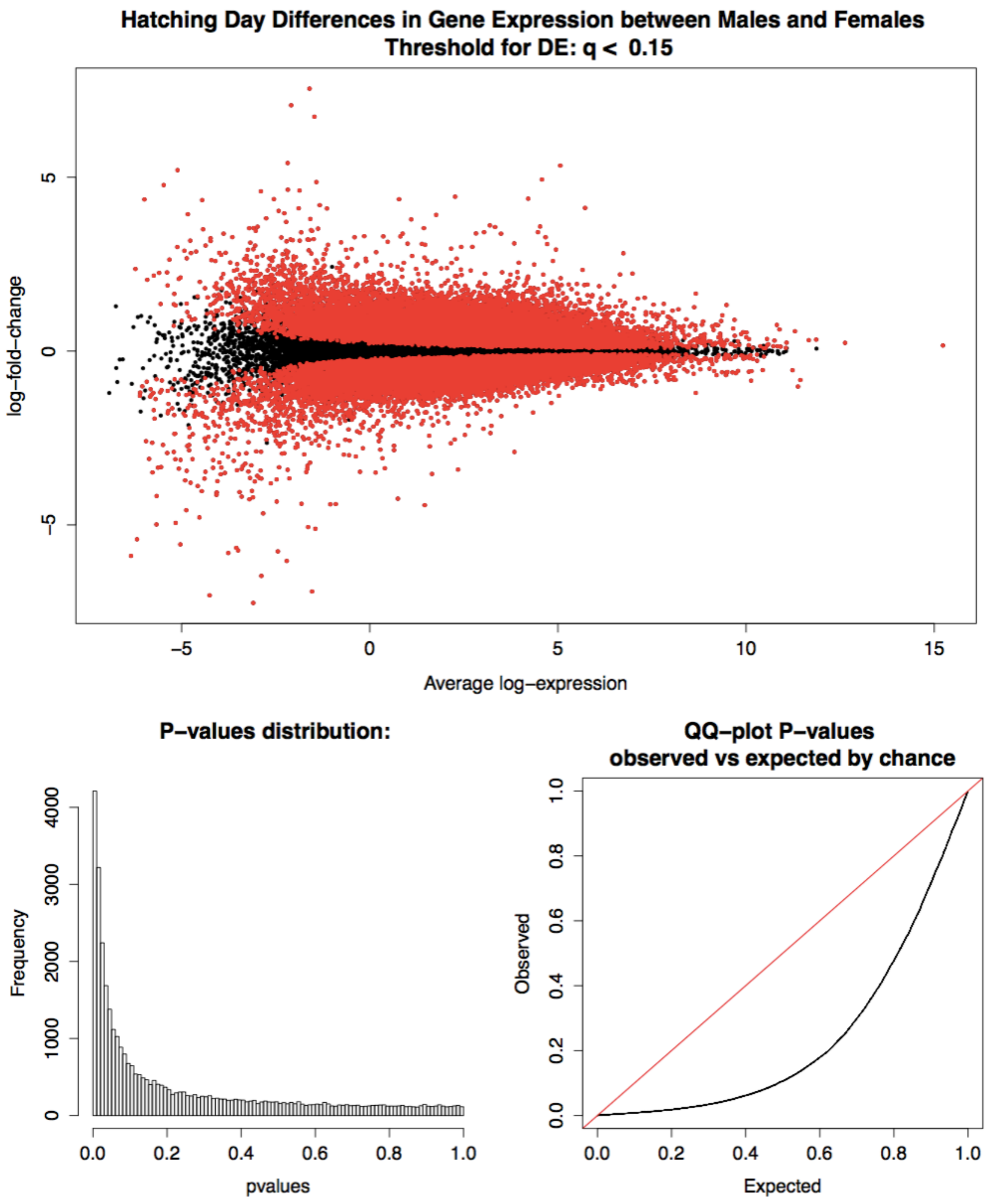

Supplementary Figure S5. Differential gene expression between control males and control females at first feeding stage (52 *dpf*). In total 1,110 genes were found differentially expressed (q<0.15) between control males and control females at first feeding stage. See Figure S4 for further explanations.

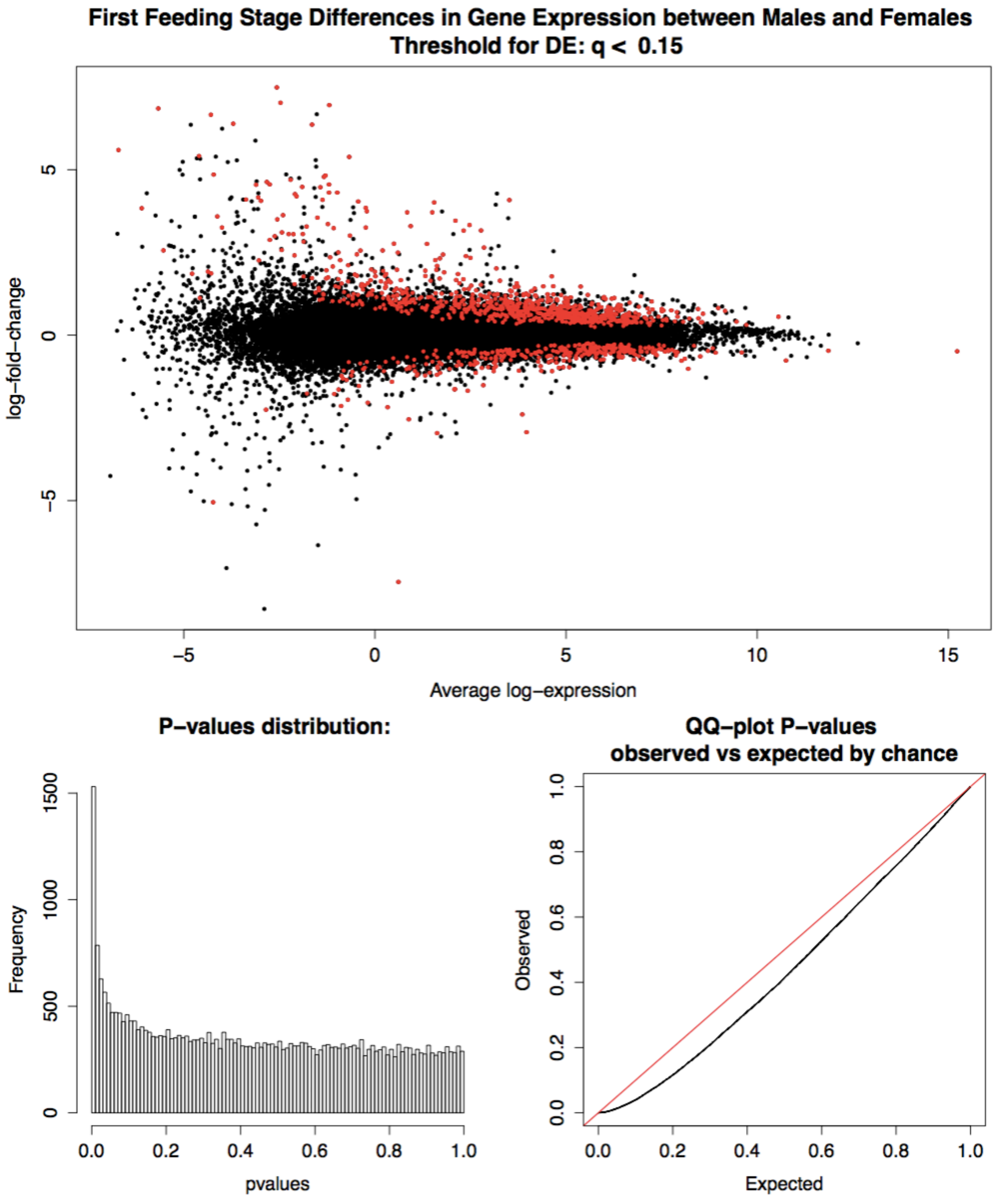

Figure S6 REVIGO treemap (Supek et al. 2011) of the 150 top gene ontology terms enriching the up-regulated genes (q<0.15) in males as compared to females at hatching (31 *dpf*).

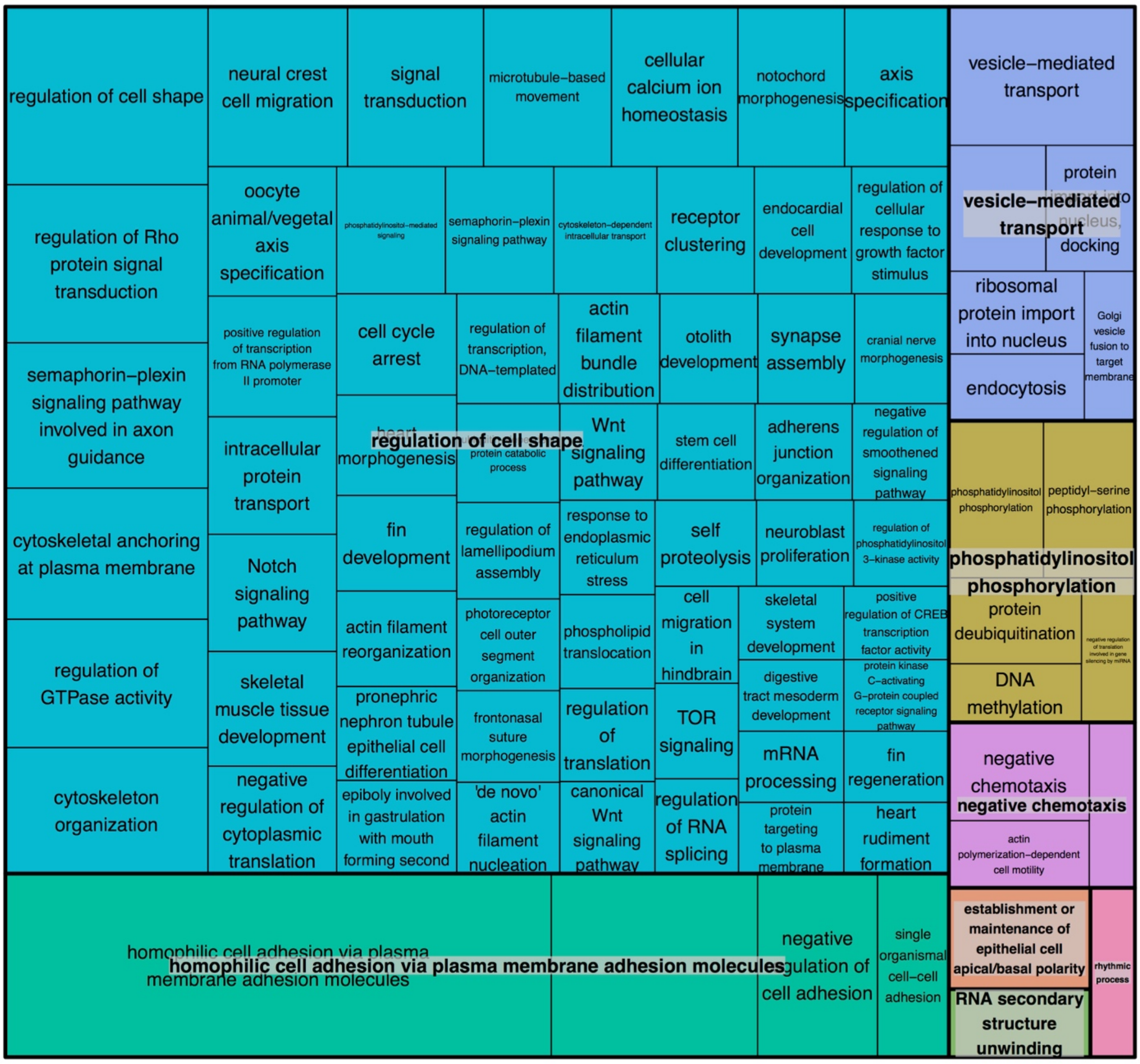

Figure S7 REVIGO treemap (Supek et al. 2011) of the 150 top gene ontology terms enriching the up-regulated genes (q<0.15) in females as compared to males at hatching (*31 dpf*).

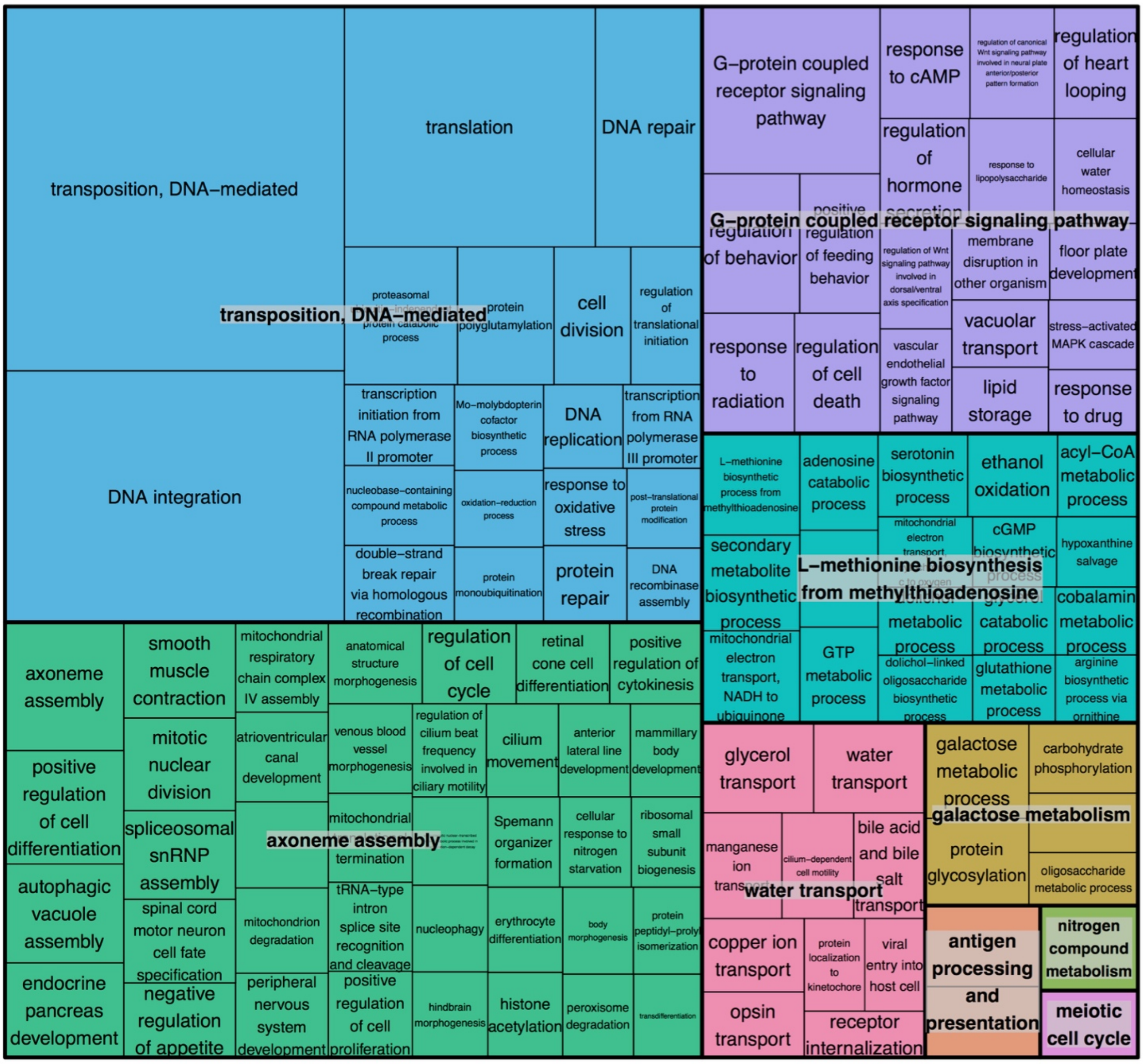

Figure S8 REVIGO treemap of the 150 top gene ontology terms enriching the up-regulated genes (q<0.15) in males as compared to females at first feeding (52 *dpf*).

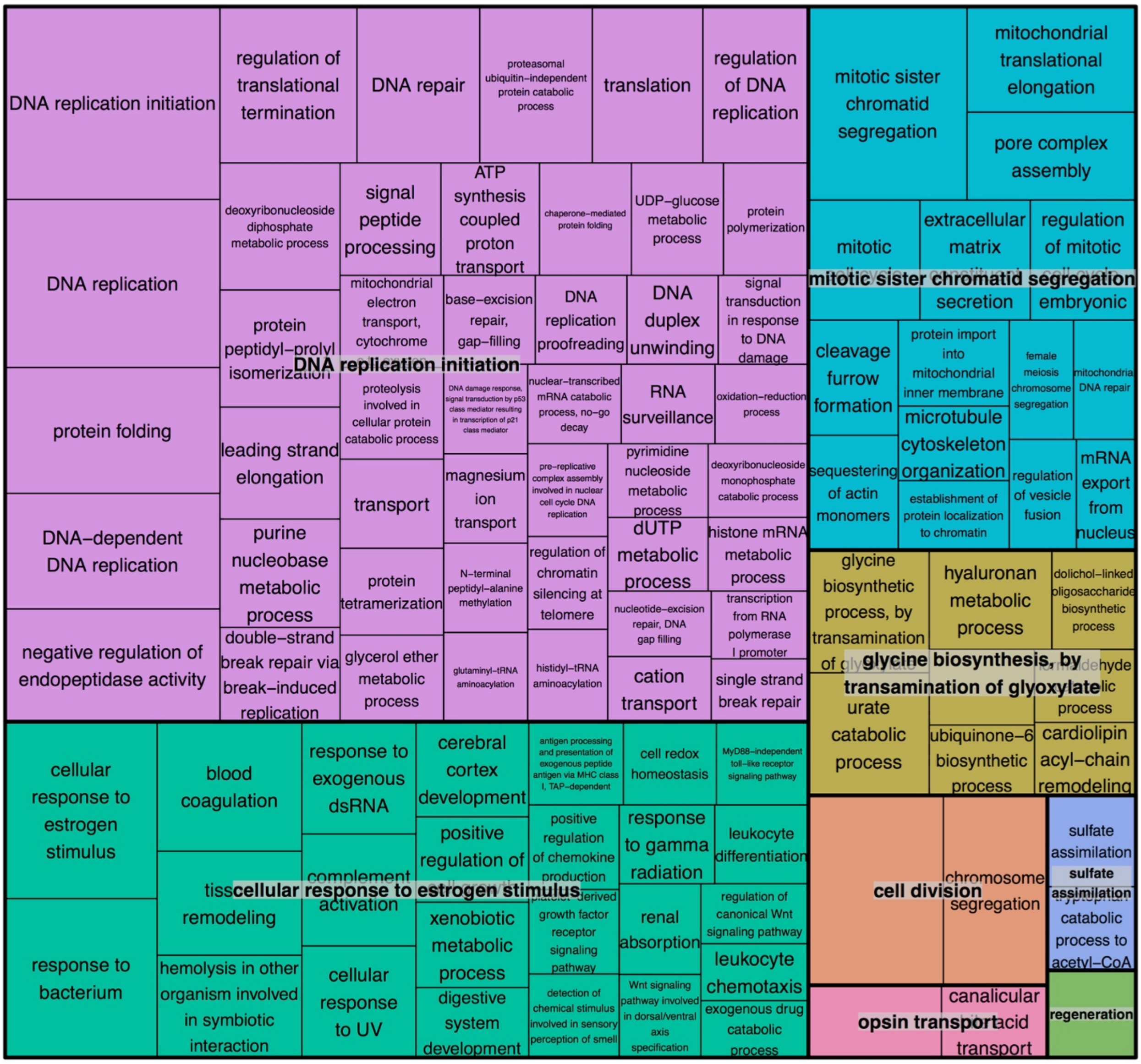

Figure S9 REVIGO treemap of the 81 top gene ontology terms enriching the up-regulated genes (q<0.15) in females as compared to males at first feeding (52 *dpf*).

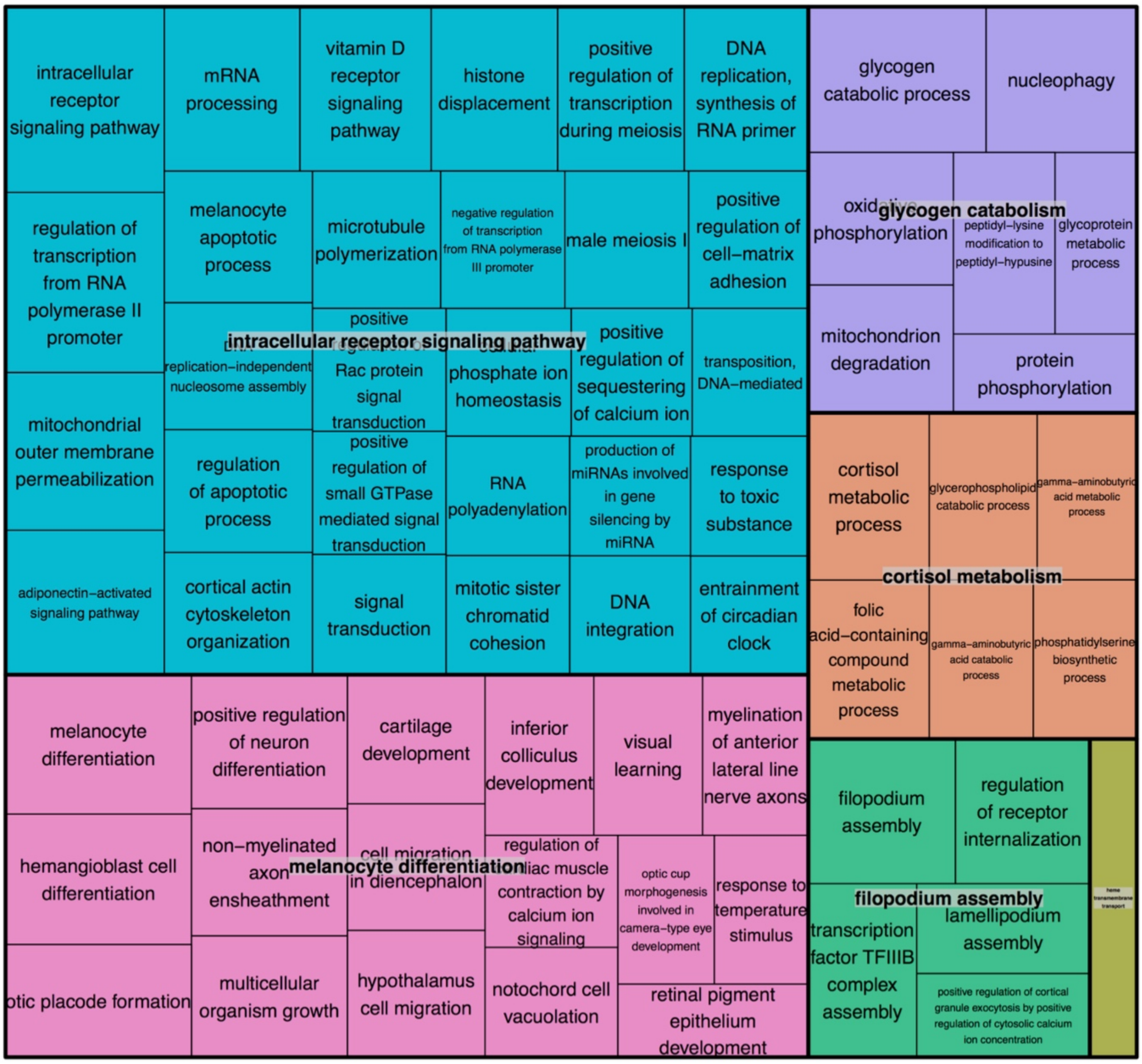

Supplement: Supplementary file 1 — Supplementary Material [file 41598_2017_14905_MOESM1_ESM.pdf]
